# A Multilevel Analysis of the Impact of Unit Tightness vs. Looseness Culture on Attitudes and Behaviors in the Workplace

**DOI:** 10.3389/fpsyg.2021.652068

**Published:** 2021-12-03

**Authors:** Daniela Di Santo, Alessandra Talamo, Flavia Bonaiuto, Cristina Cabras, Antonio Pierro

**Affiliations:** ^1^Department of Social and Developmental Psychology, Sapienza University of Rome, Rome, Italy; ^2^Faculty of Economics, Universitas Mercatorum, Rome, Italy; ^3^Department of Education, Psychology, Philosophy, University of Cagliari, Cagliari, Italy

**Keywords:** tightness vs. looseness culture, organizational outcomes, tightness, looseness, culture, work unit

## Abstract

This study examines the impact of work unit-level perceived Tightness vs. Looseness (T-L) culture on individual-level perceived stress, intention to leave, organizational deviance, job satisfaction, effort investment, and organizational commitment. Using quantitative cross-sectional data (*N*=417) collected from preexisting work units (*N*=57) in different organizations in Italy, multilevel analysis results revealed that a perceived cultural tightness at the unit level was significantly and positively related to individual-level job satisfaction, effort investment, and organizational commitment and significantly and negatively related to individual-level stress, intention to leave, and organizational deviance. The findings suggest that organizations should promote a culture of tightness to positively influence employee attitudes and behaviors. Limitations and recommendations for future research are discussed.

## Introduction

Promoting positive attitudes and behaviors among employees in the workplace is of considerable interest in organizational and psychological research. The purpose of the present work is to examine the impact of the strength of social norms and the degree of behavioral constraint (vs. latitude) in promoting positive and reducing negative attitudes and behaviors within the workplace. An adequate construct to reflect this in social systems is the Tightness-Looseness (T-L) cultural construct (Gelfand et al., 2011; Gelfand, 2018), defined as “the strength of social norms and the degree of sanctioning within societies” (Gelfand et al., 2006, p. 1226). Although a significant body of research has been developed around many cultural differences and outcomes, less attention has been paid to examining the potential impact of this cultural dimension on employees’ attitudes and behaviors.

As evidenced by growing research (Pelto, 1968; Triandis, 1989; Gelfand et al., 2006, 2011, 2017; Harrington and Gelfand, 2014; Jackson et al., 2020), the T-L framework helps explain variation between cultures with strong social norms and a low tolerance for deviance (i.e., tight) and cultures with weak social norms and a high tolerance for deviance (i.e., loose). Compared to loose cultures, tight cultures have less individual discretion, greater clarity about behavioral expectations, a limited range of appropriate behaviors, and severe sanctions for deviations from norms (Gelfand et al., 2006, 2011). Individuals’ chronic attitudes and behavior are closely connected with their cultural context; for example, individuals living in tight societies show greater caution, regulatory strength, and dutifulness (Gelfand et al., 2011).

Gelfand et al. (2006) indicate that cultural T-L can also have important effects at the organizational level in addition to societal levels. Organizational features may differ depending on whether organizations are in tight or loose contexts; more cohesive, stable and with less deviance in tight societies; less ordered and more innovative in loose societies (Gelfand et al., 2006). The T-L framework can also help explain the differences between work units (see Gelfand, 2018). In this vein, tight work units have many social norms and well-defined rules, as well as sanctions and social disapproval for inappropriate behavior; conversely, loose work units lack clearly defined norms and have a wider range of acceptable behaviors and greater tolerance of deviant behavior.

A growing body of research also links cultural T-L to various organizational outcomes, such as organizational creativity and innovativeness (Ozeren et al., 2013; Chua et al., 2015; Gedik and Ozbek, 2020), negotiation strategy (Gunia et al., 2011), perceptions of effective leadership (Aktas et al., 2016), managerial discretion (Crossland and Hambrick, 2011), expatriates’ job satisfaction (Peltokorpi and Froese, 2014), life and job satisfaction, efficacy, and exhaustion (Huang and Ren, 2017). Cultural tightness also appears to strengthen cultural values in predicting organizational outcomes (see Taras et al., 2010, for a meta-analysis).

However, to our knowledge, no research has examined the effects of perceived T-L culture on employee attitudes and behaviors in existing work units. This study aims to fill this gap by examining whether and how the perceived strength of social norms (i.e., cultural tightness) in work units affects the positive or negative attitudes and behaviors of their members. The culture of a given collective entity reflects the collective perceptions and descriptive norms that are shared by its members (Shteynberg et al., 2009). Norms exist insofar as they are “embedded in a social context that involves the shared expectations of others” (Stamkou et al., 2019, p. 948). In this research, we focus on work units because they are an appropriate level of analysis in culture research (James et al., 1990; Glisson and James, 2002; Ostroff et al., 2003) and are entities with collective properties; as such, their members typically share beliefs (i.e., norms) about expected social behaviors (Taggar and Ellis, 2007). Tight work units could promote positive effects in work units by eliminating the stress derived from uncertainty as they emphasize order and clearly defined norms. Moreover, the perception of similarity of group members is greater in tight contexts, so the risk of painful group rejection is also reduced (Uz, 2015).

The present study, therefore aims to examine, in Italy, the impact of perceived cultural tightness at the unit-level on individual-level positive and negative organizational outcomes (see Danna and Griffin, 1999, for a review), such as perceived stress, intention to leave, job satisfaction, effort investment, affective organizational commitment, and organizational deviance. Our general suggestion is that members of work units who perceive that they have clear and unambiguous rules of conduct (i.e., tightness), will put more effort into their job, feel committed to their organization, will be more satisfied with their job (e.g., Huang and Ren, 2017), feel less stressed and less willing to leave. Finally, and consistent with the suggestion of Gelfand et al. (2006), there should be less deviance from appropriate organizational behavior insofar as deviance is punished more severely. Based on the above suggestions, we formulate the hypotheses as follows:

(*H1a*) Perceived cultural tightness at the unit level will be significantly and positively related to individual-level job satisfaction.

(*H1b*) Perceived cultural tightness at the unit level will be significantly and positively related to individual-level effort investment.

(*H1c*) Perceived cultural tightness at the unit level will be significantly and positively related to individual-level affective organizational commitment.

(*H2a*) Perceived cultural tightness at the unit level will be significantly and negatively related to individual-level perceived stress.

(*H2b*) Perceived cultural tightness at the unit level will be significantly and negatively related to individual-level intention to leave.

(*H2c*) Perceived cultural tightness at the unit level will be significantly and negatively related to individual-level organizational deviance.

We tested these hypotheses in one study, which utilized employees from various Italian organizations. We examined both the linear effect and the curvilinear effect of perceived cultural tightness at the unit level on the outcome variables in question. The study is described in detail below.

## Materials and Methods

### Participants

We collected a convenience sample^1^ of 417 employees (209 men and 208 women) drawn from 16 Italian private and public organizations operating in: industrial and manufacturing sectors, financial/insurance, commercial, and health services. The sample consisted of 57 work units (minimum three maximum 26 employees, mean unit size of 11.88, *SD*=7.46), who participated in the study on a voluntary basis. Employees’ mean age was 41.56years (*SD*=10.99). About 53.5% of participants had a university degree, 40.8% had a high school degree, 1.9% had a postgraduate qualification, and 3.8% had a junior high school degree. Their mean job tenure was 13.24years (*SD*=9.92).

### Procedure

The questionnaires administered to participants included an introductory letter in which the purpose of the study was explained. Anonymity was guaranteed for all participants and their informed consent to participate in this research was appropriately obtained.

Participants first filled out the measure of perceived unit T-L culture. This was followed by self-report measures of participants’ perceived stress, turnover intentions, organizational deviance, job satisfaction, effort investment, and organizational commitment. All responses to questionnaire items were recorded on six-point scales with scale anchors 1 (*“strongly disagree”*) and 6 (*“strongly agree”*); scores were based on the mean computed across these items. Because questionnaire length was a concern, measurement of the dependent variables relied on shortened scales (three items for each scale) with items selected to be representative of the larger scale. Participants also completed demographic measures of age, sex, education, and job tenure. All materials were presented in Italian.

#### Perceived Work Unit T-L Culture (Unit-Level Variable)

Unit T-L was measured with the adapted (to the work context) version of the perceived Tightness-Looseness scale developed by Gelfand et al. (2011). The scale included 10 statements regarding the clarity and number of social norms, the degree of tolerance for norm violations, and overall compliance with social norms in the work unit. Consistent with the procedure from Gelfand et al. (2011), the respondents received the following instructions: “The following statements refer to your WORK UNIT as a whole. Please indicate whether you agree or disagree with each statement using the following scale. Note that the statements sometimes refer to '‘social norms,’ which are rules for behavior that are generally unwritten but also may be formalized/written.”

Participants were then presented with the following 10 statements: “In my work unit, there are many social norms that must be strictly followed”; “In my work unit, if someone acts in an inappropriate way, others will strongly disapprove”; “In my work unit, there are clear and well-defined rules that must be respected”; “In my work unit, it is not allowed to break the existing norms”; “In my work unit, we are pretty tolerant/indulgent if someone breaks the rules (Reversed)”; “In my work unit, if someone breaks the rules they will be punished”; “In my work unit, people generally agree upon what behaviors are appropriate versus inappropriate”; “In my work unit, people almost always comply with the existing rules”; “In my work unit, there are very clear expectations for how people should act in most situations”; “In my work unit, people have a great deal of freedom in deciding how they want to behave in most situations (Reversed).”

As the T-L scale has been adapted to the work context, we conducted an exploratory principal components analysis which showed one factor accounting for the 39.86% of the variance. Item loadings were above 0.40, with the exception of one reverse-coded item that had a loading of 0.30; this result was consistent with findings of Gelfand et al. (2011). Finally, a composite score was computed by averaging across responses to each item (*α*=0.82), where high scores indicated high levels of perceived cultural tightness in one’s work unit.

Tightness-looseness is a shared cultural construct and is most appropriately conceptualized as a group-level variable that provides a holistic view of the “social norms” (rules for behavior) that are generally present in an organizational unit. In line with previous research on T-L culture (Gelfand et al., 2011), Unit T-L was conceptualized as a group-level variable in this study. Thus, even though perceived Unit T-L was measured at the individual level, we conceptualized and aggregated this perception to the unit level, using the within-group average for each unit as a whole (i.e., the unit mean of the construct). This method assumes a bottom-up process in which lower-level properties emerge to form collective phenomena (Kozlowski and Klein, 2000). Furthermore, in our T-L culture scale, we used the referent-shift model (Chan, 1998) requiring individuals to focus on the aggregate (work unit) when answering each item scale (i.e., “In my work unit…”). This is a method frequently used in organizational climate and culture research. To justify using the unit average as an indicator of a group-level variable, it was critical to demonstrate high within-unit agreement [r_wg(j)_; James et al., 1993]. In the present study, we found that the mean r_wg(j)_ value across all units was 0.89 (*SD*=0.10), thus exceeding the recommended cutoff point of 0.70 (James et al., 1993) and providing support for data aggregation. It is notable that this value is comparable to the value (0.85) reported by Gelfand et al. (2011).

We calculated two additional aggregation statistics, ICC(1) and ICC(2). We found that the ICC(1) value exceeded the recommended cutoff of 0.06 [ICC(1)=0.30], indicating that 30% of the variance in tightness-looseness is explained by units and that the tightness-looseness scale has high inter-rater reliability. In addition, the ICC(2) value exceeded the recommended cutoff of 0.70 [ICC(2)=0.76], thus indicating that the unit level mean scores of tightness-looseness scores are highly reliable. It is likewise notable that these values are comparable to those reported by Gelfand et al. (2011).

Thus, our aggregation of work unit T-L was justified by theory and was supported by the adequate r_wg(j)_, adequate intraclass correlation indices, and significant between-groups variance, as assessed *via* a one-way ANOVA [*F*(56,360)=4.24, *p*<0.001]. Collectively, these results show that tightness-looseness is a shared, reliable construct. We also performed analysis of the normal distribution for the scores of tightness-looseness at the unit level; skewness and kurtosis were 1.047 and 2.536, respectively, thus not exceeding acceptable values.

#### Stress

Stress was measured with the following three items (*α*=0.77) derived from the Perceived Stress Scale (Cohen et al., 1983): “In the last month, I often felt nervous and stressed”; “In the last month, I often felt unable to control the important things in my life”; “In the last month, I often felt difficulties so high that I could not overcome them.”

#### Turnover Intentions

Participants responded to the following three items (*α*=0.89) derived from turnover intentions model of Mobley (1977): “I have often seriously considered finding a job elsewhere”; “I often think about leave my job”; “As soon as I have a good alternative, I will leave my organization.”

#### Organizational Deviance

Organizational deviance was self-reported by participants using the following three items (*α*=0.82) adapted from organizational deviance scale of Bennett and Robinson (2000): “I often take additional or longer breaks than is acceptable at workplace”; “I often neglect to follow my boss’s instructions”; “I often get involved on a personal matter in working hours.”

#### Overall Job Satisfaction

Job satisfaction was assessed with the following three-item scale (*α*=0.92) derived by Brayfield and Rothe (1951) measure: “Most days I am enthusiastic about my work”; “I feel fairly satisfied with my present job”; “I find real enjoyment in my work.”

#### Effort Investment

Participants responded to the following three items of the effort measure developed by Brown and Leigh (1996): “When there is a job to be done, I devote all my energy to getting it done”; “I work at my full capacity in all of my job duties”; “Few of my colleagues put in more hours weekly than I do.” The reliability analysis showed a Item-Total Correlation close to 0 for this last item, which was therefore omitted from subsequent analyses. The reliability of the effort measure therefore resulted in *α*=0.81.

#### Organizational Commitment

Organizational commitment was measured with the following three items (*α*=0.68) from the Affective Commitment Subscale of Organizational Commitment Scale developed by Meyer et al. (1993). A widespread approach to organizational commitment in literature (*cf.* Allen and Meyer, 1990) considers commitment as an affective or emotional attachment to the organization, its goals and values, beyond its purely instrumental worth (e.g., continuance and normative commitment). The following items were administered: “I would be very happy to spend the rest of my career with this organization”; “I do not feel a strong sense of belonging to my organization (Reversed)”; “This organization has a great deal of personal meaning for me.”

## Results

Analyses were conducted using SPSS version 25. Table 1 contains a summary of descriptive statistics and zero-order bivariate correlations. Both perceived cultural tightness at the individual and unit levels were positively and significantly correlated with individual-level job satisfaction, effort investment, and organizational commitment; both perceived cultural tightness at the individual and unit levels were negatively and significantly correlated with individual-level perceived stress, turnover intentions, and organizational deviance. Furthermore, as shown in Table 1, job satisfaction, effort investment, and organizational commitment were significantly and positively intercorrelated, and were significantly and negatively correlated with perceived stress, turnover intentions, and organizational deviance. Finally, the latter constructs were significantly and positively intercorrelated.

**Table 1:** Descriptive statistics and variable intercorrelations.

	*M* (*SD*)	1	2	3	4	5	6	7	8
1. Ind. tightness	3.89 (0.82)	1							
2. Unit tightness	3.89 (0.52)	0.630^***^	1						
3. Job Satisfaction	4.43 (1.12)	0.373^***^	0.306^***^	1					
4. Stress	2.94 (1.21)	−0.189^***^	−0.243^***^	−0.360^***^	1				
5. Turnover Int.	2.84 (1.46)	−0.209^***^	−0.205^***^	−0.528^***^	0.498^***^	1			
6. Effort	4.81 (0.93)	0.363^***^	0.335^***^	0.384^***^	−0.271^***^	−0.302^***^	1		
7. Org. Commitment	4.22 (1.15)	0.375^***^	0.313^***^	0.577^***^	−0.338^***^	−0.620^***^	0.383^***^	1	
8. Org. Deviance	2.16 (1.10)	−0.319^***^	−0.240^***^	−0.295^***^	0.361^***^	0.329^***^	−0.505^***^	−0.314^***^	1

*N*=417; Team *N*=57; Ind. tightness=perceived cultural tightness at the individual level; Unit tightness=perceived cultural tightness at the unit level; Stress, perceived stress; Turnover Int., turnover intentions; Effort, effort investment; Org. Commitment, organizational commitment; and Org. Deviance, organizational deviance.

*** *p*<0.001.

We used multilevel modeling to test our hypotheses on the impact of perceived cultural tightness at the unit level. In each analysis (one for each outcome variable), we entered as fixed the main effect of unit cultural tightness at the *group* level. In this analysis, age, sex, education, and job tenure were entered as control variables. Finally, only the intercept was a random effect, entered at the unit level. Therefore, our presentation of the results focuses on the fixed effects. This multilevel model was analyzed using restricted maximum likelihood (REML) estimation.

Results generally show that the control variables have no impact on the relationships between the main variables of the study and thus the discussion of their effects is omitted.

More importantly, after controlling for age, sex, job tenure, and education, multilevel analysis results revealed that a perceived cultural tightness at the unit level was significantly and positively related to individual-level job satisfaction [*b*=0.62, *SE*=0.13, *t*=4.692, *p*<0.001, 95% CI (0.356, 0.886)], effort investment [*b*=0.56, *SE*=0.11, *t*=4.922, *p*<0.001, 95% CI (0.334, 0.790)], and organizational commitment [*b*=0.65, *SE*=0.12, *t*=5.200, *p*<0.001, 95% CI (0.399, 0.903)]; and significantly and negatively related to individual-level perceived stress [*b*=−0.59, *SE*=0.14, *t*=− 4.321, *p*<0.001, 95% CI (−0.869, −0.317)], intention to leave [*b*=−0.46, *SE*=0.21, *t*=− 2.202, *p*=0.032, 95% CI (−0.878, −0.040)], and organizational deviance [*b*=−0.55, *SE*=0.14, *t*=− 4.065, *p*<0.001, 95% CI (−0.822, −0.282)].

We also introduced a quadratic term into the multilevel model to account for possible curvilinear effect; the curvilinear effects for each of the outcomes were not significant (all *p*s>0.143), hence this notion was not further investigated.

## Discussion

The main goal of the present study was to examine the effects of perceived cultural tightness at the work unit level on various positive and negative outcomes at the individual level. Confirming our hypotheses (see Table 2), the results have shown that perceived cultural tightness at the unit level was positively associated with job satisfaction (H1a), effort investment (H1b), and organizational commitment (H1c), whereas it was negatively associated with perceived stress (H2a), turnover intentions (H2b), and organizational deviance (H2c). These results are quite consistent with previous literature suggestions and findings (e.g., Huang and Ren, 2017), and open many possible scenarios.

**Table 2:** Summary of hypothesis testing.

Hypothesis	Supported?	Finding
H1a	Yes^**^	Perceived cultural tightness at the unit level is significantly and positively related to individual-level job satisfaction
H1b	Yes^**^	Perceived cultural tightness at the unit level is significantly and positively related to individual-level effort investment
H1c	Yes^**^	Perceived cultural tightness at the unit level is significantly and positively related to individual-level affective organizational commitment
H2a	Yes^**^	Perceived cultural tightness at the unit level is significantly and negatively related to individual-level perceived stress
H2b	Yes^*^	Perceived cultural tightness at the unit level is significantly and negatively related to individual-level intention to leave
H2c	Yes^**^	Perceived cultural tightness at the unit level is significantly and negatively related to individual-level organizational deviance

* *p*<0.05., and

** *p*<0.001.

### Theoretical and Managerial Implications

Having knowledge of the effect of a shared T-L can potentially improve management policy. Based on the findings of this research, maintaining a tight culture could be a good practice for leaders and employers to foster positive attitudes in their work units. One possible application is to put effort into building a tight environment to increase positive attitudes, while decreasing negative attitudes. However, it cannot be said that a tight culture is always recommended for work units. Much can depend on workers’ characteristics, whether they have a tight or loose mindset and adhere to social norms to a greater or lesser extent. Furthermore, work units can be heterogeneous, as in multinationals or large companies, and consist of individuals with different cultural characteristics (e.g., from tight and loose cultures) that influence their familiarity with rigid or relaxed rules; for example, restricted leeway may be less motivating for employees from a loose culture and could negatively affect their attitudes in the workplace. Therefore, HR managers and leaders should pay attention to individual and cultural characteristics of the work unit employees. Interestingly, a “person-group fit” could be realized (Pierro et al., 2015); a “fit” between the T-L culture of a work group and the tight-loose mindset of employees, as well as other individual characteristics (e.g., self-regulation orientations; Higgins, 2012), can promote positive attitudes and behaviors. We recommend that future research explore this aspect.

Finally, in organizations in loose rather than tight societies, employees may have more positive workplace attitudes in loose culture work units. Interestingly, in the debate on whether freedom over constraints favors the welfare of society or vice versa, scholars (Harrington et al., 2015) found that both excessive freedom and excessive constraint are costly and a balance between permissiveness and constraint produces optimal national outcomes. Therefore, we examined possible curvilinear effects, but they were not significant in our sample. It is possible that the type of organizations to which the surveyed work units belong may have accounted for this result. It is also possible to find different relationships between cultural tightness and these outcome variables in a more creative/design/high tech setting; this should be profitably investigated by future research. Moreover, we do not yet know what happens when work units are threatened (i.e., in conditions that threaten its survival), but following national findings (e.g., Harrington and Gelfand, 2014), a tightening of norms should help them coordinate to survive.

### Limitations

While this research may help examine real working environments, there are noteworthy limitations. One limitation derived from the cross-sectional design and that cultural tightness was measured and not manipulated in this study, which limits causal inferences in the relations between cultural tightness and the various outcomes. Further research should profitably examine these effects using longitudinal designs or manipulating cultural tightness in work units. In addition, positive and negative outcomes were measured as self-reported data. This can cause a response bias that is common to research fields, where self-reported data are used (Rosenman et al., 2011). We thus cannot eliminate the possibility that biased self-perceptions may have affected our data. Although for some specific variables (e.g., job satisfaction) self-reported measurement is the most used (e.g., Brayfield and Rothe, 1951), this limitation can be addressed using other sources, for example, behaviors at workplace. It would also be important to use other and more objective indicators of effectiveness at work (e.g., job performance).

However, we can cautiously conclude that common method/source bias is unlikely to have affected our results, as unit-level aggregate T-L perception had an impact on individual attitudes. Furthermore, the use of mean unit perceptions tends to attenuate both random variance in individual responses and systematic differences (such as personality, background, and previous experiences) that could contaminate individual perceptions (James et al., 1990; Seibert et al., 2004).

### Suggestions for Future Research

Creativity at work deserves particular attention among the outcomes that can be further examined, given the previous results on cultural T-L and creativity (Harrington and Gelfand, 2014; Chua et al., 2015) showing that tight states have lower creativity than loose states (Harrington and Gelfand, 2014). We might expect tight work units to have little creativity, but it would be possible to find specific moderators of this effect (see also Gedik and Ozbek, 2020). For example, according to regulatory focus theory (e.g., Higgins, 2012), individuals’ self-regulation is distinguished in a self-regulatory focus on eager advancement (i.e., promotion focus) and a self-regulatory focus on vigilant maintenance (i.e., prevention focus), which are shown to have opposite effects on creativity (Friedman and Förster, 2001), positive and negative, respectively; thus, in work units composed of employees with a high prevention focus, a tight culture should inhibit creativity, while in work units composed by employees with a high promotion focus, a loose culture should foster creativity. These relationships are worth exploring for future research.

As mentioned above, it is also important to emphasize that the culture of the work unit is likely to be influenced by the culture of the organization in which it is embedded, and, more generally, by the culture of the country in which it is based (see also Ozeren et al., 2013). In a sense, a unit is an organizational mini culture embedded in a larger one, hence reflecting the values and norms of the larger organization. It is therefore recommended to extend the examination of these effects to the organizational level. At the same time, organizational cultures are embedded in their country’s culture (Gelfand et al., 2006) and previous research has shown that some organizational cultures reflect national cultural characteristics (Ozeren et al., 2013). It is, therefore, worth examining the extent to which the T-L culture of the country (Gelfand et al., 2011) in which the organization, or work unit, is located can interact with the cultural T-L perception shared by the organization, or work unit, in promoting positive or negative outcomes in the workplace. Further research is recommended, but the present findings may help improve understanding of the T-L effects in real working environments.

## Data Availability Statement

The raw data supporting the conclusions of this article will be made available by the authors, without undue reservation.

## Ethics Statement

The studies involving human participants were reviewed and approved by Ethical Committee of the Department of Social and Developmental Psychology, Sapienza University of Rome. The patients/participants provided their written informed consent to participate in this study.

## Author Contributions

All authors contributed to the conception and design of the study. DS wrote the first draft of the manuscript. AP executed the study and analyzed the data. All authors contributed to the article and approved the submitted version.

## Funding

This work received funding from the Sapienza Ateneo Scientific Research 2018 (Principal Investigator: Prof. Antonio Pierro), Protocol. No. RG118163F3F6F0FE.

## Conflict of Interest

The authors declare that the research was conducted in the absence of any commercial or financial relationships that could be construed as a potential conflict of interest.

## Publisher’s Note

All claims expressed in this article are solely those of the authors and do not necessarily represent those of their affiliated organizations, or those of the publisher, the editors and the reviewers. Any product that may be evaluated in this article, or claim that may be made by its manufacturer, is not guaranteed or endorsed by the publisher.

## Footnotes

^1^Various organizations were contacted and given a standard explanation concerning the purpose of the research; the data was collected in cases where organizations and employees voluntarily agreed on research participation.
